# Releasing incompatible males drives strong suppression across populations of wild and *Wolbachia*-carrying *Aedes aegypti* in Australia

**DOI:** 10.1073/pnas.2106828118

**Published:** 2021-10-04

**Authors:** Nigel W. Beebe, Dan Pagendam, Brendan J. Trewin, Andrew Boomer, Matt Bradford, Andrew Ford, Catherine Liddington, Artiom Bondarenco, Paul J. De Barro, Joshua Gilchrist, Christopher Paton, Kyran M. Staunton, Brian Johnson, Andrew J. Maynard, Gregor J. Devine, Leon E. Hugo, Gordana Rasic, Helen Cook, Peter Massaro, Nigel Snoad, Jacob E. Crawford, Bradley J. White, Zhiyong Xi, Scott A. Ritchie

**Affiliations:** ^a^School of Biological Sciences, University of Queensland, Brisbane, QLD, Australia, 4072;; ^b^Commonwealth Scientific and Industrial Research Organisation, Brisbane, QLD, Australia, 4102;; ^c^College of Public Health, Medical and Veterinary Sciences, James Cook University, Smithfield, QLD, Australia, 4878;; ^d^Australian Institute of Tropical Health and Medicine, James Cook University, Smithfield, QLD, Australia, 4878;; ^e^QIMR Berghofer Medical Research Institute, Brisbane, QLD, Australia 4029;; ^f^Creative Communicator, Cairns, QLD, Australia 4870;; ^g^Verily Life Sciences, South San Francisco, CA 94080;; ^h^Department of Microbiology and Molecular Genetics, Michigan State University, East Lansing, MI 48824

**Keywords:** vector control, biological control, incompatible insect technology, arbovirus vector, *Aedes aegypti*

## Abstract

Releasing sterile or incompatible male insects is a proven method of population management in agricultural systems with the potential to revolutionize mosquito control. Through a collaborative venture with the “Debug” Verily Life Sciences team, we assessed the incompatible insect technique (IIT) with the mosquito vector *Aedes aegypti* in northern Australia in a replicated treatment control field trial. Backcrossing a US strain of *Ae. aegypti* carrying *Wolbachia w*AlbB from *Aedes albopictus* with a local strain, we generated a *w*AlbB2-F4 strain incompatible with both the wild-type (no *Wolbachia*) and *w*Mel-*Wolbachia Ae. aegypti* now extant in North Queensland. The *w*AlbB2-F4 strain was manually mass reared with males separated from females using Verily sex-sorting technologies to obtain no detectable female contamination in the field. With community consent, we delivered a total of three million IIT males into three isolated landscapes of over 200 houses each, releasing ∼50 males per house three times a week over 20 wk. Detecting initial overflooding ratios of between 5:1 and 10:1, strong population declines well beyond 80% were detected across all treatment landscapes when compared to controls. Monitoring through the following season to observe the ongoing effect saw one treatment landscape devoid of adult *Ae. aegypti* early in the season. A second landscape showed reduced adults, and the third recovered fully. These encouraging results in suppressing both wild-type and *w*Mel-*Ae. aegypti* confirms the utility of bidirectional incompatibility in the field setting, show the IIT to be robust, and indicate that the removal of this arbovirus vector from human-occupied landscapes may be achievable.

## Significance

With over 40% of humans at risk from mosquito-borne diseases such as dengue, yellow fever, chikungunya, and Zika, the development of environmentally friendly mosquito-control tools is critical. The release of reproductively incompatible male mosquitoes carrying a *Wolbachia* bacterium can drive mating events that kill the eggs. Through replicated treatment and control experiments in northern Australia, regular releases of *Aedes aegypti* males infected with *a Wolbachia* from *Aedes albopictus* was shown to drive strong population suppression in mosaic populations of wild-type (no *Wolbachia*) and *w*Mel-*Wolbachia*–carrying *Ae. aegypti*. In a demonstration of bidirectional incompatibility between different *Wolbachia* strains in the field, we also demonstrate that one season’s suppression experiment can also show an ongoing effect into the following season.

Mosquitoes transmit parasites and viruses that infect hundreds of millions of humans annually. Globally, one invasive species—*Aedes aegypti* (Linnaeus)—is responsible for the greatest transmission of arboviruses, causing diseases including yellow fever, dengue, chikungunya, and Zika (1). The paucity of effective vaccines for most of these diseases and observed concerns for both growing insecticide resistance and insecticide’s adverse effects on other beneficial species have prompted renewed interest in species-specific biological control methods (2).

Releasing sterile or incompatible male insects en masse is one method of insect population control currently under development (3). The sterile insect technique (SIT) uses the release of sterile male insects that search and mate with wild females to prevent subsequent production of offspring. The SIT has been successfully used to control various insect pest species (4), including the New World screwworm fly (5), the tsetse fly (6), the Mediterranean fruit fly (7), and the apple codling moth (8). Previous SIT technologies have been hampered by their need for radiation or chemical sterilents, which can compromise male fitness, requiring the release of more sterile males to compensate for their fitness decline (9, 10). Furthermore, the development of genetic modification to generate sterile male *Ae. aegypti* mosquitoes has been hampered by perceived environmental risks as well as strong regulatory issues and community acceptance (11).

The first applications of SIT to mosquitoes encountered mixed successes during the 1970s and ’80s (12), as it proved difficult to produce sufficient numbers of competitive sterile males to suppress natural populations. More recently, elegant transgenic approaches have generated sterile male mosquitoes for the malaria vector *Anopheles gambiae* (13), the dengue vector *Ae. aegypti* (3, 14), and the Asian tiger mosquito *Aedes albopictus* (15). Releases of transgenic sterile *Ae. aegypti* by Oxitec Ltd in Grand Cayman have demonstrated an effective reduction of these mosquitoes (16).

Reproductively incompatible males can now be produced using a maternally inherited gram-negative endosymbiotic bacterium *Wolbachia pipientis* (17, 18). Many of these strains carry a cytoplasmic incompatibility (CI) phenotype, where males of one *Wolbachia* strain can be reproductively incompatible with females that do not carry the strain; thus, females lay eggs that fail to hatch (18). This scenario provides an opportunity to reassess the SIT using what is now termed the incompatible insect technique [IIT (19)]. The SIT is generally more effective when females are not released (20), as sterile female insects can still damage crops, transmit disease, or simply distract sterile males from searching out wild mates. In the case of mosquito SIT and IIT, releasing males means only releasing mosquitoes which do not bite, have fewer risks, and encourage community acceptance.

The primary aim for this study was to demonstrate the effectiveness of an *Ae. aegypti* IIT in a replicated treatment control study using males infected with a *Wolbachia w*AlbB strain from *Ae. albopictus*. Australia’s northern Queensland towns have endemic populations of *Ae. aegypti* (21), some of which have recently been transformed to carry the *w*Mel *Wolbachia* strain (transfected from *Drosophila melanogaster*) in order to reduce arbovirus transmission risk (22): this phenotype provides arbovirus-blocking properties (23). We hypothesized that by selecting isolated towns and suburbs in tropical northern Queensland, we could demonstrate strong *Ae. aegypti* suppression over one season. By monitoring over the following season, we also sought to test whether suppression during one season would have a subsequent impact on populations the following season. At the outset of this study, all *Ae. aegypti* populations in the study site were wild type (no *Wolbachia*). However, during preparations for our IIT experiment—and at the request of the Queensland state health authority—all experimental landscape populations were transformed by high levels of *w*Mel*-*infected *Ae. aegypti* released by Eliminate Dengue (now the World Mosquito Program [WMP]) (24). Subsequently, we released *w*AlbB2-infected males into a mosaic population of both wild type and *w*Mel-*Ae. aegypti* to induce incompatible mating. We observed strong suppression over 20 wk, with bidirectional incompatibility in a field setting—and evidence of an ongoing suppression effect lasting into the next season.

## Results

### Incompatible Mosquito Strain Development.

The *w*AlbB2-F4 *Ae. aegypti* strain was developed through four backcrosses of Innisfail *Ae. aegypti* wild-type males with females from a US WB2 strain already transfected with *Wolbachia* strain *w*AlbB from *Ae. albopictus*. This was provided by the Z.X. laboratory at Michigan State University. Four rounds of backcrossing *w*AlbB2 females with local Queensland males were implemented to achieve >90% genome transfer (25), while providing the strain with a preadapted north Queensland genetic background. CI of this *w*AlbB2-F4 strain was demonstrated to be 100% to both the wild-type Cairns females and the *w*Mel-carrying *Ae. aegypti* that had been established in the experimental landscapes (see *SI Appendix*, Fig. S1 for results). Approval to release the *w*AlbB2-F4 strain from Australian quarantine was provided on October 2017 by the Australian Department of Agriculture and Water Resources (DAWR), and the strain was then transported to the James Cook University (JCU) Mosquito Rearing Facility (MRF), which is a 1.5-h drive from the experimental landscapes. Approval to release *w*AlbB2-F4 males as a biopesticide was subsequently provided in November 2017 by the Australian Pesticide and Veterinary and Medicines Authority (PER7250).

### Release Landscape, Mosquito Rearing, and Delivery.

**Fig. 1.**
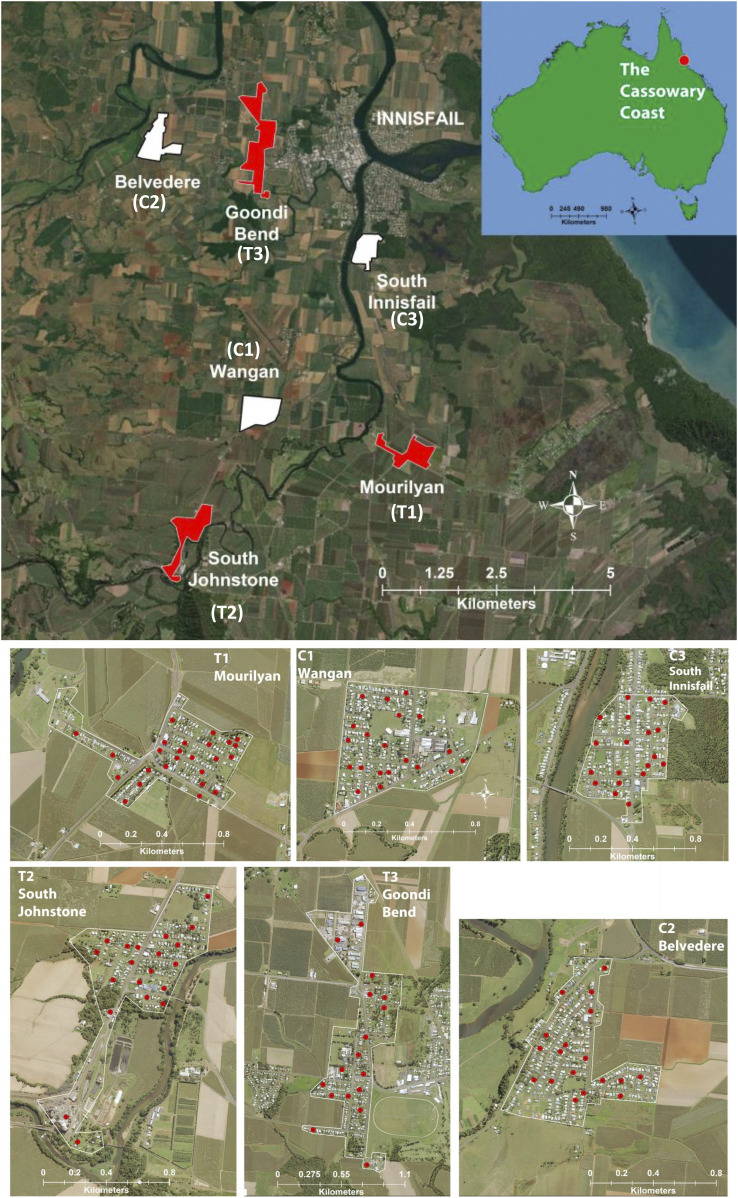
Field trial sites in the Northern Cassowary Coast region. Control landscapes are white, and treatment landscapes are red. Urban landscapes are isolated by extensive sugar cane, and banana plantations and the proximity of all landscapes to each other are show in the upper panel, with more-detailed images of the three treatment (T1 to T3) and the three control landscapes (C1 to C3) in the lower panel. Red dots indicate positions of paired BGS traps and ovitraps used for monitoring.

The landscapes selected for the replicated treatment/control experiment are shown in Fig. 1 and described in Table 1. The three treatment landscapes (T1 to T3) were composed of 237 to 256 sites (buildings/houses) and included the towns of Mourilyan (T1, −17.5812°, 146.0414°), South Johnstone (T2, −17.5972°, 145.9963°), and the isolated outer suburb of Goondi Bend (T3, −17.52143°, 146.0104°), which is part of the larger town of Innisfail (−17.5227°, 146.0278°; Fig. 1). The three control landscapes (C1 to C3) comprised between 170 to 307 sites and were monitored along with the treatment landscapes: adults were collected using BG Sentinel (BGS), and eggs were collected in ovitraps, with collections in all landscapes occurring twice per week. All landscapes showed slightly different spatial topography with regard to shape and site structure (Fig. 1). In addition, the wild-type *Ae. aegypti* populations of these landscapes had recently been transformed to a mostly *w*Mel-carrying population (24).

**Table 1.** Summary of the treatment and control landscapes and males released

Areas	Treatment sites	Area	Traps	Trap /Ha	Human population*	Males released	Total males/m^2^
		Ha					
T1 Mourilyan	256	44	18	0.41	527	943439 1150609^†^	208 254^†^
T2 South Johnstone	237	65	20	0.31	413	878725 907693^†^	164 168^†^
T3 Goondi Bend	232	85.5	16	0.19	594	859307 890212^†^	103 106^†^
C2 Wangan	307	52	20	0.38	641		
C1 Belvedere	327	48	20	0.42	907		
C3 South Innisfail	179	33	20	0.61	506^‡^		
Totals	1,538	327.5	114		3,505		

* Australian Bureau of Statistics 2016.

^†^ Including pretrial test releases in November to December 2018.

^‡^ Population slightly less than stated, as some marginal streets were not monitored.

Males were generated for release at JCU in Cairns through mass rearing and a two-step sex separation process developed by Verily Life Sciences and described in an earlier publication (26). The sexually dimorphic smaller male pupae were hand sieved for size to remove ∼95% of female pupae prior to emergence into an adult sex sorter. Further adult separation was estimated to achieve an accuracy of over 1 female in 1 million males sorted (26). Males were released using a modified van-based delivery system developed by Verily, including a computer-controlled Google maps–based automated mosquito-release system (26). A predetermined number of males were puffed onto the road edge via holes in the passenger side of the vehicle as the vehicle was moving. Males were delivered to the treatment sites on Monday, Wednesday, and Friday mornings each week. The frequency of releases was used to maintain overflooding pressure in light of the short lifespan of male *Ae. aegypti* in the field—estimated at an average lifespan of 1.6 or 1 to 3 d as observed through our own mark–release–recapture studies (27) and other studies (28, 29).

### The *Ae. aegypti* Population-Suppression Experiment.

**Fig. 2.**
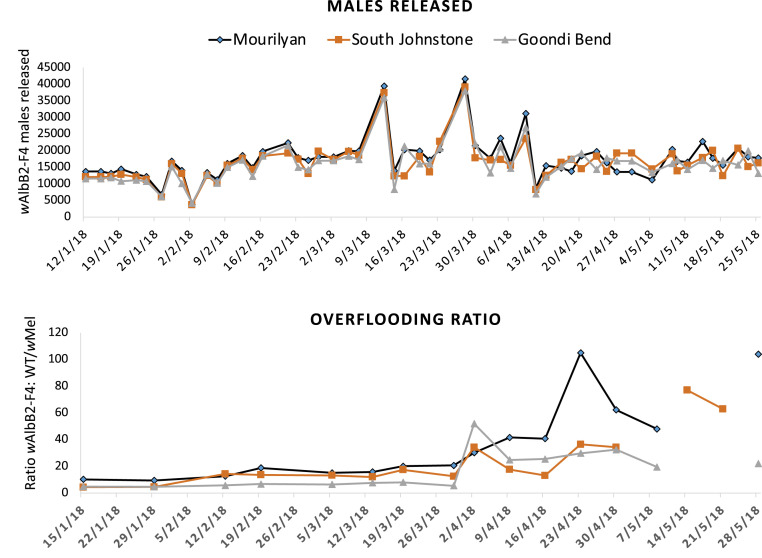
Male release numbers and male overflooding ratios. The upper panel shows the *w*AlbB2-F4 release numbers into the treatment landscapes. During November to December 2017, 11 test releases were performed in into T1 (Mourilyan) over 3.5 wk, and two test releases were run into T2 and T3 (South Johnstone and Goondi Bend) over a single week. Then after a gap of 5 wk, continuous releases began on January 12, 2018, with 54 releases over 20 continuous weeks that ended on May 25, 2018. Fluctuations in release numbers reflect manual mass rearing productivity variation early in the releases. The lower panel shows the calculated overflooding ratio determined from the number of *w*AlbB2-F4 males divided by the total number of wild-type and *w*Mel males in the traps. Gaps in the graphs during May 2018 manifest when no wild-type or *w*Mel males were collected and overflooding ratios could not be calculated.

Through November and December 2017, test releases of *w*AlbB2-F4 males were run to finetune and test the mosquito rearing, sorting, and delivery equipment. Testing included 11 releases over 4 wk in Mourilyan (T1) and two releases over 1 wk in both South Johnstone and Goondi Bend (T2 and T3, Fig. 2). Although our initial goal was to achieve an overflooding ratio of ∼15:1 (*w*AlbB2-F4 males to field males), limitations to our manual rearing and sex sorting resulted in the release of ∼50 *w*AlbB2-F4 males per site into each treatment landscape at the outset of the experiment. Releases into the treatment landscapes occurred on Friday, Monday, and Wednesday starting on Jan 12, 2018. Overflooding ratios of ∼5:1 (T2 and T3) and 10:1 (T1), with release numbers and male overflooding ratios, are summarized in Fig. 2. Laboratory production of males fluctuated early in the releases as we adapted to the mass rearing system (Fig. 2). Occasionally, bacterial bloom–driven die offs at the larval stage led to lower release numbers, which was particularly evident on Jan 27 and March 2 (Fig. 2). Overflooding ratios were determined through BGS trapping of adult males, with PCR used to discriminate *w*AlbB2-F4 from wild-type and *w*Mel *Ae. aegypti*. From the outset of releases into the treatment landscapes, only T1 achieved an overflooding ratio consistently greater than 10:1 over the initial 2 wk, which increased as suppression ensued. Overflooding ratios in T2 began below 5:1 then increased to above 10:1 by week three. Landscape T3 also began below 5:1 but did not breach 10:1 until 9 wk into releases (April 3). By this time, all landscapes were exhibiting high overflooding ratios (greater than 30:1) as adult suppression continued. The jump in overflooding ratio was assisted by larger male releases on March 12 and 28, where numbers could be increased by greater larval rearing productivity and higher deliveries into landscapes (Fig. 2). Additionally, the dramatic change in overflooding ratios in all treatment landscapes observed 12 wk into the releases likely also reflects a local hurricane (Tropical Cyclone Nora) that moved through all landscapes toward the end of March (March 24 to 28, 2018). Elevated overflooding ratios were detected in the April 3 collections for T2 and T3; these decreased over subsequent weeks but remained elevated thereafter.

**Fig. 3.**
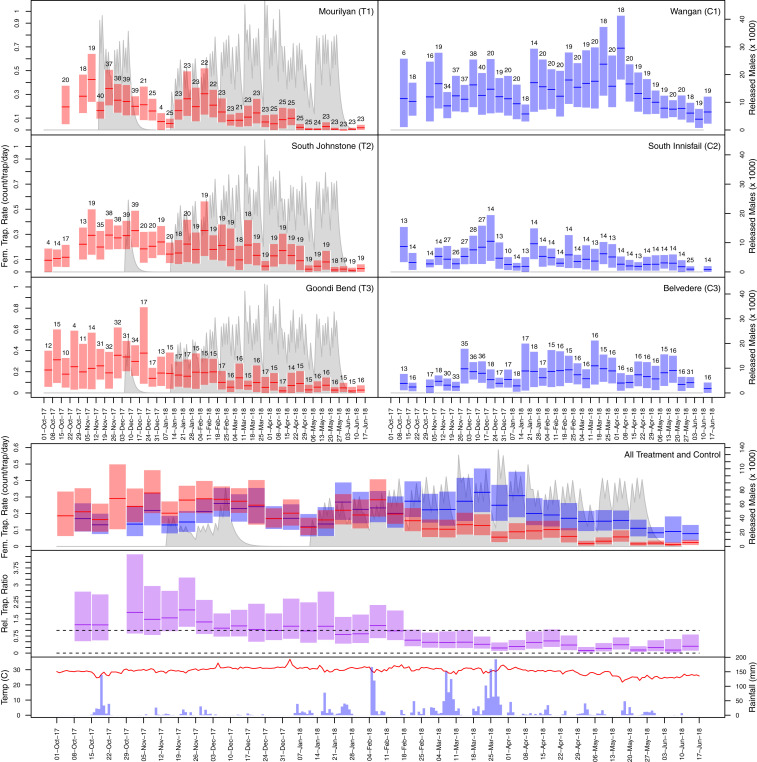
Population suppression summary for *Ae. aegypti* adults collected in BGS traps. The upper panels shows adult weekly mean BGS trapping rate and bootstrapped 95% CIs, with treatments shown as red bars (T1 to T3) and controls shown in blue bars (C1 to C3). The numbers above the bars represent the number of measurements contributing to the trapping rate estimate and includes pre-experiment test releases. The gray lines show the expected number of living released *w*AlbB2-F4 males assuming 30% death rate per day. Test releases through November and December 2017 (11 into T1 and two into T2 to T3) were followed by a 5-wk hiatus, and then 55 releases over 20 wk (three per week) were sustained into T1 to T3 (January 12 to May 25). The lower panels show aggregated treatment and control plots for the suppression experiment (red T1 to T3 and blue C1 to C3). The population suppression of *Ae. aegypti* is shown in the second bottom panel as relative trap ratio of treatments to controls in purple. The dashed lines delineate values of 1 and 0, between which the treatment landscapes are lower than in the controls (i.e., suppression). In the bottom panel, temperature is tracked by the red line, with rainfall shown as blue bars.

**Fig. 4.**
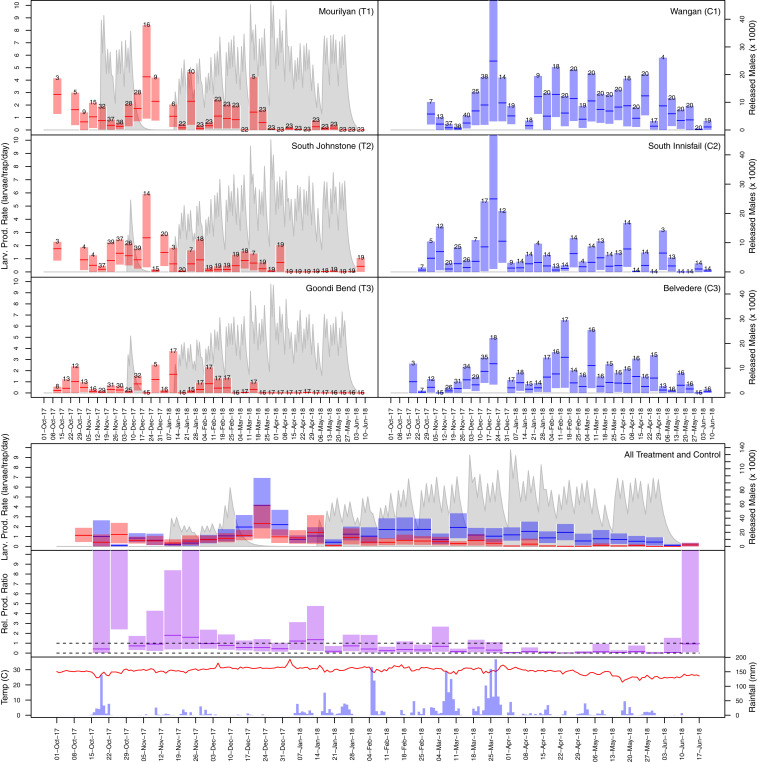
Larval productivity of *Ae. aegypti* in paired ovitraps. The upper panels show adult weekly mean larval productivity rate and bootstrapped 95% CIs, with treatments shown as red bars (T1 to T3) and controls shown in blue bars (C1 to C3). The numbers above the bars represent the number of measurements contributing to the trapping rate estimate. The gray lines show the expected number of living released *w*AlbB2-F4 males assuming 30% death rate per day. Test releases through November and December 2017 (11 into T1 and two into T2 to T3) were followed by a 5-wk hiatus, and then 55 releases over 20 wk (three per week) were sustained into T1 to T3 (January 12 to May 25). The lower panels show aggregated treatment and control plots for larval productivity (red T1 to T3 and blue C1 to C3). *Ae. aegypti* larval productivity is shown below that as relative trap ratio of treatments to controls in purple. The dashed lines delineate values of 1 and 0, between which the treatment landscapes are lower than in the controls (i.e., suppression). In the bottom panel, temperature is tracked by the red line with, rainfall shown as blue bars.

Test releases were performed mostly in T1 prior to the experiment starting in order to assess rearing and release equipment; thus, T1 received more males per hectare than T2 or T3 over the 20-wk release period. In this, T1 received 27% more males per hectare than T2 and 100% more males per hectare than T3. If the pre-experiment test releases are included (11 releases into T1 and two releases into T2 and T3), T1’s males per hectare increased to 51 and 140% more than T2 and T3, respectively (see Figs. 3 and 4, T1 to T3 for pre-experiment test releases).

### Changes in the Adult *Ae. aegypti* Population.

Adult *Ae. aegypti* were monitored through an array of BGS traps maintained at a density of one to two per neighborhood block (Fig. 1), with trap density per hectare provided in Table 1. The BGS adult collections in treatment and control landscapes are shown in Fig. 3, *Upper*. Suppression, determined by comparing the number of adults collected across the three treatment landscapes (T1 to T3) to the control (C1 to C3) landscapes over the duration of the IIT experiment (January 12 to May 25, 2018), can be seen in Fig. 3, *Lower*. The test releases that ended 5 wk prior to the primary experiment resulted in adult numbers starting with parity between treatment landscapes (T1 to T3) and control landscapes (C1 to C3, Fig. 3). All populations increased again in early January 2018 before the suppression’s effect was visible—in week four, the treatment and control populations started to diverge (Fig. 3, *Lower*). Adult numbers in control landscapes stayed relatively constant through much of the season, while the trapping rate in treatment landscapes continued to decline with larval productivity acting as a leading indicator of suppression (Fig. 4). In early April 2018, the *Ae. aegypti* population suppression (measured as a relative female trap ratio of adult treatment to control counts/trap/day) exceeded 0.3 (70%), and adult numbers in controls declined through April after Tropical Cyclone Nora traversed all landscapes in late March (24–28). Population suppression exceeded 0.2 (>80% suppression) over several weeks, with controls falling and the mosquito season ending prematurely. Significant downward trends in treated populations relative to control populations were identified using a time series of log-relative population. Here, linearity under a log transform would be expected if the treated population was undergoing exponential decay (population suppression) using the nonparametric Theil–Sen estimator (30). Summaries of nine pairwise comparisons exhibited negative trends, indicating that population suppression was evident (Table 2). Eight of the nine comparisons showed statistically significant downward trends at the 0.05 significance level. As we are making simultaneous hypothesis tests, it is common to use a method to control either the familywise error rate (FWER) or the false discovery rate (FDR). Using the Bonferroni correction (31) to control the probability of making one or more Type-I errors to less than 0.05, we found that five out of nine tests were significant. The method of Benjamini and Hochberg (32) for controlling the FDR to be less than 0.05 showed eight of the nine hypothesis tests were significant. Controlling the FDR has become commonplace since its development, as it provides a useful alternative to the conservative Bonferroni correction, which has diminished statistical power as the number of hypothesis tests grows in number. We report both for transparency in Table 2. Overall, this analysis provided strong evidence that the treated landscapes showed evidence of population suppression relative to the control landscapes

**Table 2.** Trends in log relative population time series using all possible pairwise comparisons of treatment and control landscapes

Treatment	Control	Sen’s Slope	*P*	Significant BF	Significant B-H
T1 Mourilyan	C1 Wangan	−0.130	3.11 × 10^−5^	Yes	Yes
T1 Mourilyan	C2 Belvedere	−0.125	8.01 × 10^−5^	Yes	Yes
T1 Mourilyan	C3 South Innisfail	−0.116	1.14 × 10^−3^	Yes	Yes
T2 South Johnstone	C1 Wangan	−0.077	2.98 × 10^−5^	Yes	Yes
T2 South Johnstone	C2 Belvedere	−0.080	7.94 × 10^−4^	Yes	Yes
T2 South Johnstone	C3 South Innisfail	−0.057	1.20 × 10^−2^	No	Yes
T3 Goondi Bend	C1 Wangan	−0.058	1.01 × 10^−2^	No	Yes
T3 Goondi Bend	C2 Belvedere	−0.053	1.39 × 10^−2^	No	Yes
T3 Goondi Bend	C3 South Innisfail	−0.034	1.28 × 10^−1^	No	No

Columns labeled as “Significant BF” and “Significant B-H” show whether the *P* is statistically significant under the Bonferroni or Benjamini–Hochberg corrections, respectively

### Changes in *Ae. aegypti* Larval Productivity.

Larval productivity was measured over the experimental period as the number of larvae emerging from egg collections on ovitraps [Fig. 4 (33)]. Larval productivity was used in preference to egg hatch rate due to the difficulty of assessing viable eggs on ovitrap sticks that were either semicollapsed (potentially viable) or collapsed (nonviable), as a result of a mating from *w*Mel-*Ae. aegypti* male and wild-type females (CI-impacted eggs are nonviable). Suppression within each treatment landscape was initially noisy, with larval productivity peaks seen through December 2017 coinciding with a period of no rainfall, making ovitraps a preferential oviposition site. Differences between treatment and control landscapes became evident after 2 wk of continuous releases when aggregated (Fig. 4). The larval productivity rate decreased to low levels through the last 10 wk of releases, with productivity in T2 and T3 being virtually undetectable. Male releases ended on May 25, and collections over the subsequent week saw larval productivity in traps rebound in T2 in a way that correlated with reduced rainfall in early June.

### Ongoing Effects of the *Ae. aegypti* Population-Suppression Trial.

**Fig. 5.**
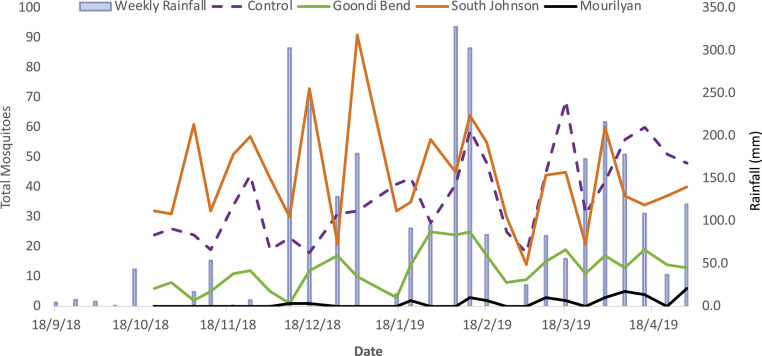
*Ae. aegypti* IIT suppression ongoing effect through the following season. Adult *Ae. aegypti* were monitored using BGS traps collected weekly over 26 wk through the following season in all three treatment landscapes (T1-Mourilyan, T2-South Johnstone, and T3-Goondi Bend) and one control lanscape (C1-Wangan). Rainfall is indicated by blue bars. Treatment 1 (Mourilyan) showed the strongest suppression effect across the following season with very few adults detected, most of which were collected late in the season. Landscapes T2 (South Johnstone) appeared to fully recover, and T3 (Goondi Bend) showed a reduced population through the following season.

We followed the carryover effect of the suppression experiment (January to May 2018) through the following season with a weekly monitoring program that ran for 27 wk (from spring Oct 10, 2018 through summer to April 30, 2019). Only one control landscape (C1-Wangan) was monitored, with all landscapes utilizing 13 BGS traps per landscape. The C1 control had collected a total of 262 *Ae. aegypti* adults by week 10 and 999 adults by week 20. The C1 and T1 landscapes’ spatial topology appeared most similar in size, structure, proximity (Fig. 1 and Table 1), and *Ae. aegypti* productivity observed prior to male releases in Dec 2017 (Fig. 3). In the following season, no adult *Ae. aegypti* were collected in T1 until week 8, with only two adults collected by week 10 and 35 *Ae. aegypti* trapped by week 27 (Fig. 5). Comparing adult captures between C1 and T1 the following season, we observed a 99% suppression effect at week 10 and 97% suppression effect by week 27 (Fig. 5). The T3 landscape (Goondi Bend) had a 70% reduction (81 adults) at week 10 and ∼63% by week 27 (348 adults). Only T2 (South Johnstone) showed no suppression effect in the following season and rebounded strongly, with 440 and 1,202 *Ae. aegypti* adults at weeks 10 and 27, respectively.

The 2017 to 2018 IIT intervention appeared to have minimal effect on the relative proportion of the *w*Mel-*Ae. aegypti*–transformed population the following season, with high percentages of *w*Mel-*Ae. aegypti* detected in all landscapes monitored (*SI Appendix*, Fig. S2). Fluctuations in the proportion of wild-type and *w*Mel-*Ae. aegypti* observed through the summer coincided with a 1-wk heatwave that began Nov 25, 2018. During the peak of summer, *w*Mel-*Ae. aegypti* adult levels decreased, though rarely dropping below 60% of the population (*SI Appendix*, Fig. S2).

## Discussion

Traditional vector control has largely failed to control the globally invasive mosquito *Ae. aegypti* due to widespread insecticide resistance and lack of political will and thus resources (34, 35). Here, we demonstrate the effectiveness of using incompatible male *Ae. aegypti* releases into isolated landscapes containing both wild-type and *w*Mel-*Ae. aegypti*. We observed population suppression above 80% when the aggregate of the three treatment landscapes are compared to the aggregate of the three controls. We also demonstrated the effectiveness of the technique in driving bidirectional incompatibility in a field setting and observed the effect of this developing vector control method lasting well into the following season, with one of the three treatment landscapes showing over 97% suppression 11 mo later.

In total, 3 million incompatible male *Ae. aegypti* carrying the *Ae. albopictus* lineage B *Wolbachia* (*w*AlbB2-F4) were released into three isolated north Queensland urban communities over a 20-wk period. Adult mosquito suppression was detectable after week four and was preceded by substantial declines in larval productivity. The strategy to release males three times a week, driven by our mark–release–recapture work (27) and other studies (28, 29), took into account the short lifespan in the field of *Ae. aegypti* males to effectively maintain male overflooding ratios. The selection of isolated treatment and control landscapes separated by sugar cane and banana plantations reduced the *Ae. aegypti* reinvasion rate into the emptying niche, with strong suppression still observable into the following season in one landscape (T1) and a reduced population in a second (T3). Importantly, we did not detect any *w*AlbB2-F4 females or larvae in traps, suggesting the Verily sex-sorting system was highly effective. The Verily sex-sorting pipeline claims a female contamination rate of 1:900 million males (26), which is far beyond the potential for human error when morphologically separating adult female from males in BGS trap collections. The nondestructive DNA-extraction method utilized meant anomalies such as misidentifying trapped adults (i.e., a released male being called a female after BGS collections were processed) could be reassessed by checking the morphology of carcasses left after the DNA extraction.

In demonstrating the novelty of CI to a population of both wild-type and *w*Mel-*Ae. aegypti* in the field, we could generate strong population suppression, and this intervention had little effect on the *w*Mel-*Ae. aegypti* infection level in the field. When populations responded the following season, the percentage of *w*Mel-*Ae. aegypti* within all landscapes returned to their former high levels of over 90%. This indicates that release of *w*AlbB males suppresses *w*Mel and wild-type populations to the same extent, without disproportionately inducing sterility in one population more than another. This result was encouraging, as it was previously unknown if the suppression experiment would push the unstable equilibrium frequency of *w*Mel-*Ae. aegypti* to below 30%, where it could potentially drop out of the population (22), resulting in an increase of the risk of arbovirus transmission. In fact, high fluctuations in *w*Mel-*Ae. aegypti* proportions were observed after a heatwave in late November 2018. This is consistent with similar observations during the same heatwave in Cairns (75 km north of our study site), where *w*Mel-*Ae. aegypti* larvae frequency and *w*Mel *Wolbachia* adult densities varied considerably (36).

Field trials of the IIT are now accelerating, with Verily Life Sciences seeing encouraging outcomes in their area-wide *Ae. aegypti* IIT control experiments in California (Fresno-Clovis) (26). Verily has developed innovative digital support technologies for the automated mass rearing of larvae, female pupal removal and adult sex sorting, and a sophisticated vehicle-based adult delivery system. In California, males were delivered daily via this vehicle-based system into treatment landscapes immediately from the mosquito season’s outset, achieving high overflooding ratios of between 47:1 and 202:1 (26). Males also carried *w*AlbB, and trials achieved over 90% suppression between replicated treatment and control sites (26). The proximity of these trial treatment landscapes to adjacent *Ae. aegypti* populations meant females migrated back into the emptying niches, and 100% suppression or elimination could not be achieved despite the high overflooding pressure. In contrast, we began releases when landscapes were already productive and several months into the mosquito season. While we observed a male *w*AlbB2-F4 overflooding ratio to wild-type/*w*Mel males of between 5:1 and 10:1, all treatment populations collapsed at similar rates. This outcome suggests that high overflooding rates are not necessarily more effective, and there may be an optimal ratio that maximizes the efficiency of incompatible male interventions. Most importantly, we delivered males into isolated populations, where the effect of suppression could be measured the following season. The strong suppression observed through the following season in our T1 site suggests that *Ae. aegypti*’s removal from isolated urban landscapes is possible. However without barriers or boundaries, invasion back into the empty niche can occur rapidly as was observed in the American Verily study (26). The question of why the isolated T2 population recovered the following wet season requires further investigation. Nonetheless, it appears that *Ae. aegypti*’s biology—high human feeding preference and container inhabiting ecology—can constrain this mosquito to urban landscapes, presenting potential isolated populations (villages, towns, and cities) that would encourage the IIT as a tool to population removal.

Trials of the IIT on the Asian tiger mosquito, *Ae. albopictus,* in China and Italy have also been encouraging (37–39). The larger study in China demonstrated strong suppression of the species over three seasons by targeting relatively isolated riverine islands in Guangzhou, China (37). Authors developed a triple-*Wolbachia Ae. albopictus* strain (HC) also carrying *w*Pip from *Culex pipiens* that showed 100% cytoplasmic incompatibly to the native strain. Using an initial overflooding ratio of ∼5:1, they detected a 65% reduction in adults in the first season. Here, the release numbers were limited because manual inspection for adult females after mechanical sex separation occurred to prevent unintentional release of contaminated females and establishment of HC strain in the field that could cause the failure of the trial. A pupal-stage irradiation treatment step was then developed for seasons two and three that allowed higher male releases numbers, providing 83 to 94% reductions in the adult population. Like the Verily study in California, movement of *Ae. albopictus* back into the treatment landscapes likely prevented higher suppression levels. Unlike *Ae. aegypti* males, which only live a few days postrelease (28, 29), *Ae. albopictus* males appear to survive for up to 2 wk (38), perhaps due to their avid sugar-feeding behavior (40, 41). Thus, in this system, less-frequent releases may still be as effective for suppression of the population.

Although the reduced fitness of some *Wolbachia* strains and potential colony effects may call for higher ratios at the outset of releases, systematic backcrossing to the wild population is recommended to maintain optimum fitness and fecundity (42, 43). As the *Wolbachia* bacterium is maternally inherited (through the egg), wild males under constant selection for fitness can be backcrossed with *Wolbachia*-carrying females, and all offspring will carry the bacterium. This backcrossing to maintain male fitness is less complicated than maintaining a homozygous-dominant modification such as is required for the release of insects carrying a dominant lethal system (44). Indeed, the short lifespan of the males may mean that insecticide-sensitive males may also be effective in insecticide-resistant populations, although this could not be tested because Queensland *Ae. aegypti* populations remain insecticide sensitive (45).

Preseason monitoring through October to December 2017 showed that treatment landscapes were more productive than the controls (Fig. 3). However, test releases in treatment landscapes, which stopped 5 wk prior to the official experiment starting, brought the aggregate adult numbers close to parity for the outset of the experiment. The subsequent population suppression proved highly effective across the mosaic of wild-type and *w*Mel-carrying *Ae. aegypti*, demonstrating the utility of bidirectional incompatibility in a field setting.

On monitoring the landscapes the following season, we observed very few *Ae. aegypti* in T1 (Mourilyan) despite setting traps in areas that had previously been highly productive. To achieve this level of suppression, the standing egg bank in T1 must have been depleted, rendering the population of *Ae. aegypti* potentially vulnerable to elimination. Despite T1 being a highly productive landscape at the outset of the study, the suppression effect through the following season showed undetectable levels of *Ae. aegypti* for the first 8 wk despite abundant rainfall before and during the collection period (Fig. 5). This conclusion is reasonable, as T1 is well isolated from other *Ae. aegypti* populations and received a higher density of males (per m^2^) than T2 and T3. In this, T1 received 20% more *w*AlbB2-F4 males than T2 and 50% more than T3. Moreover, T1 received additional pre-experiment releases due to equipment testing, which ended 5 wk prior to the experimental period in January 2018 (nine releases over 4 wk). We cannot discount the possibility that traps were not sampling the population comprehensively, as the trapping array was not designed for the possibility of elimination. Nonetheless, these traps had provided ample numbers of mosquitoes during the previous season, before, and during the primary experimental period. For future elimination trials, we suggest a broader array of trapping through the landscape, and attempts should also be made to classify adults as local or immigrants with high-resolution genomic screening tools now available (46, 47). The transformation of the field population to a *w*Mel-*Ae. aegypti* strain (Cairns genetic background) may compromise the use of population genetics assessment to detect immigrants, as the genetic sweep of the maternally inherited *Wolbachia* could alter the population genetic structure of the population in these isolated landscapes.

In summary, selection of release landscapes was primarily driven by the unique opportunity to use small, isolated towns and suburbs supporting wild *Ae. aegypti* populations in northern Queensland. By the time releases began, the *Ae. aegypti* population contained both wild and *w*Mel phenotypes, allowing us to observe the bidirectional incompatibility of *w*AlbB2-F4 male mosquitoes with *w*Mel-*Ae. aegypti* females. We found that initial overflooding ratios of 5:1 were highly effective and could provide production efficiencies with a lower risk of releasing *Wolbachia*-carrying females if efficient sex-sorting technologies were not available. As such, the presence of different strains of *Wolbachia*-*Ae. aegypti* exhibiting bidirectional incompatibility could provide an effective means of removing strains that accidently established in a landscape. However, the lower fitness of these *Wolbachia* females compared to the wild females already in the landscape (48)—along with their potential deficiencies such as insecticide resistance common in many field populations (49)—may permit some female leakage through the system early in releases; this would need to be reduced when a niche starts to empty (50). Alternatively, rotating *Ae. aegypti* strains with different incompatible *Wolbachia* strains could mitigate female establishment. If *w*AlbB2-F4 were to have established during our experiments, the *Ae. aegypti* population would still have carried both a flavivirus- and alphavirus-blocking phenotype (51), with stronger transmission-blocking capability to the *w*Mel strain (52), plus higher thermal tolerances than the *w*Mel strain that currently deployed for disease control (53).

Limitations to incompatible- and sterile male–release technologies may arise in large continual landscapes, as population suppression beyond 95% can be difficult to achieve as females from outside the treatment landscape may establish back into the emptying niche, as was seen in previous studies (26, 37). Continual monitoring is also required to detect these migration events, including monitoring after an eradication has been reached. Optimization of suppression and eradication activities in larger landscapes will come from theoretical models that explore different control scenarios eventually improving the cost effectiveness of these male-release techniques (50, 54). Efficiencies in mass rearing, sex separation, and field delivery will also develop as this technology matures and becomes more automated (26). For example, releasing *Ae. aegypti* males three times a week to maintain male overflooding levels can be logistically difficult. If the short-lived *Ae. aegypti* males could be prepared so that they survive for one additional day in the field, the release frequency could move from three times a week to twice a week, resulting in a significant efficiency gain.

*Ae. aegypti* is a highly urbanized tropical arbovirus vector currently extant in a subset of north-Queensland towns. Our results suggest the removal of this globally invasive tropical species is now more plausible with the IIT. In the Australian context, the effects of climate change and our adaptation to it may increase the risk of this species establishing (and re-establishing) in towns and cities. In particular, the warming and dryer climate in much of Australia means our adaptation to drought-proof towns and cities with rainwater tanks are potentially providing ideal thermally buffered niches for the larvae, and the species may once again exist beyond the tropical biology restriction observed in the 20th century (21, 55). Additionally, the more temperate-adapted *Ae. albopictus* already inhabits islands only 30 km from the northern-Australian mainland. The current control strategy is primarily insecticide driven (56), and this is unlikely to be sustainable over time. With ecological niche models suggesting this temperate species will survive alongside much of populated Australia (57), an IIT for both species will provide critical new tools to remove both these invasive species from Australia.

## Conclusion

The utility of using *Wolbachia*-carrying mosquitoes as a vector control tool appears to be twofold, as they can be deployed for either population replacement or population removal. Population replacement requires the release of both male and female *Wolbachia*-infected mosquitoes and can reduce virus transmission in *Ae. aegypti* populations by reducing the virus-amplification potential of the female mosquito: encouraging field trials have been completed in this space (58, 59). Population removal occurs through the release of only males: this drives population suppression and provides opportunities for population removal, reducing virus transmission by removing the mosquito. The IIT has the potential to revolutionize the management of vector populations, and our work here demonstrates the IIT’s utility as an area-wide and environmentally friendly solution for the suppression of *Ae. aegypti* populations.

*Ae. aegypti* males appear short lived in the field, but releasing incompatible males three times a week for 20 wk into established populations of mostly *w*Mel-infected *Ae. aegypti* could deliver strong suppression with modest initial overflooding ratios of 5:1 to 10:1. This outcome was encouraging; however, perhaps the more encouraging outcome came in the following season, where we saw a population-suppression carryover effect in two of the three treatment landscapes. One of these landscapes did receive additional *w*AlbB males prior to the experiment starting, which may have aided in emptying the niche of *Ae. aegypti* that carried through to the following mosquito season due to the restricted migration back into the landscape. It would be interesting to see what two seasons of IIT in these isolated landscapes may have achieved in terms of understanding what eradication may look like. A clearer idea of what eradication looks like and how we get there may now come from further analysis of the different outcomes in our three treatment sites.

With increasing incursions of both *Ae. aegypti* and *Ae. albopictus* across the world, developing tools such as the IIT can be important in the battle to contain and remove invasive mosquitoes that vector human pathogens, as the males are well-evolved to find females at a variety of locations, including oviposition sites and human hosts (60). As traditional vector control fails to contain new incursions and established populations, the idea of rolling back populations from towns and cities may now be feasible through more-automated mass production and strategic delivery methods such as these.

## Materials and Methods

### Landscape Selection and Monitoring Strategy.

Urban landscapes that include small towns and remote suburbs throughout the Cassowary Coast in north Queensland provided ideal landscapes to study *Ae. aegypti* population suppression. These urban landscapes are isolated by means of extensive sugarcane and banana plantations in the region reducing potential movement between *Ae. aegypti* populations. After 2 y of surveys using gravid *Aedes* traps (GATs), to confirm the presence of *Ae. aegypti*, three treatment landscapes and three control landscapes were selected for the trial. Landscapes were chosen based on mosquito abundance and relative isolation in order to mitigate mosquito migration. Treatment landscapes included the towns of Mourilyan, (T1, 256 premises), South Johnstone (T2, 237 premises), and a secluded suburb of Innisfail, Goondi Bend (T3, 232 premises). Three control landscapes (monitoring only) included Wangan (C1, 307 premises), Belvedere (C2, 327 premises), and South Innisfail (C3, 179 premises). See Table 1 and Fig. 1 for more detail. The towns of Cairns (75 km north) and Townsville (220 km south) had previously been transformed to an *Ae. aegypti*-*w*Mel–carrying population by Eliminate Dengue (24)—now the WMP. All selected landscapes were transformed from wild-type *Ae. aegypti* to *Ae. aegypti*-*w*Mel by the WMP 6 mo prior to IIT releases starting (24).

Mosquito monitoring involved a trapping design utilizing paired BGS traps (https://eu.biogents.com/bg-sentinel/) to obtain adults (species, male/female counts) and ovitraps consisting of a small black 500-mL bucket, containing 250 mL tap water with a lucerne pellet [for the microbiome olfactory attractant (33)]. A labeled large medical tongue depressor (6-in flat stick) was placed against the inside of the bucket for oviposition. Both the BGS and ovitraps were placed at a landscape density of one to two traps per neighborhood block (Fig. 1) with traps density per hectare estimated. Traps were serviced twice per week, and a trapping digital data stream based on QR codes was used to track the adults and eggs collected. In the laboratory in Cairns, the adults were identified to species and sex by stereo microscope, placed in 96-well plates containing 100 µL 96% ethanol, and sent to University of Queensland (UQ) in Brisbane for molecular analysis. Eggs from ovitrap sticks were embryonated for 2 d on the sticks and transferred to JCU in Cairns (1.5-h drive). Eggs were then hatched, and larvae were reared until third instar (3 to 5 d, depending on density), where they could be reliably separated from local *Ae. notoscriptus* (Skuse) species. Larval productivity was represented by the number of third-instar larvae per ovitrap.

### Incompatible Mosquito Strain Development.

The *Ae. aegypti* IIT strain was generated by backcrossing an imported US *Ae. aegypti* WB2 strain (from Prof. Zhiyong Xi at Michigan State University), previously transinfected with *Ae. albopictus* B lineage *Wolbachia* (61), under import permit 0000799042. All work (importation, maintenance, back-crossing, and characterization) was carried out at the Mosquito Control Laboratory, QIMR Berghofer, under strict quarantine conditions governing the development of the wAlB2-F4 strain and its subsequent release to JCU. Four backcrosses to a Cairns background colony were performed in duplicate by placing two adult males (Cairns) with five US *Ae. aegypti-w*AlbB2 virgin females (separated at pupal stage), resulting in the “*Ae aegypti w*Alb*B2*-F4”strain. Presence of maternally inherited *w*AlbB2 *Wolbachia* was confirmed by PCR (see *Molecular Identification of Field-Collected Samples*), and CI to wild-type and *w*Mel *Ae. aegypti* was shown through mating experiments using *w*AlbB2-F4 males and wild-type and *w*Mel females. A report on the strain was circulated through key stakeholders for consultation and included the Australia’s Department of Environment, DAWR, Australian Pesticides and Veterinary Medicines Authority (APVMA), and Queensland Health. Permission to release the strain from quarantine was granted in November 2017 (DAWR reference 2017/074), and approval to use the strain as a released biopesticide was granted by the APVMA (PER 84077), also in November 2017. The strain was sent to the JCU’s MRF for mass rearing under a material transfer agreement from QIMR Berghofer.

### Assessment of *w*AlbB2-F4 CI and Fecundity.

Cross-mating experiments were conducted to assess CI in both *w*AlbB2-F4 and *w*Mel mosquitoes using four strains of *Ae. aegypti: w*AlbB2 (Michigan), *w*AlbB2-F4 backcross, Cairns, wild-type, and *w*Mel strains. Larvae were raised as per standard procedure (see *Mass Rearing, Sex Sorting, and Field Delivery*). Due to sexual size dimorphism at the pupal stage, pupae were sex sorted by eye to collect virgin males and females for each of the strains. *w*AlbB2-F4 CI experiments involved cross-mating between virgin females of the four different strains whereas *w*Mel suppression experiments involved pairing of virgin females of the different strains with virgin *w*Mel males. Using 2- to 3-d-old mosquitoes, each cup had one virgin male with three virgin females. Control cups involved crosses of the same strain, and all cups were provided with 10% sucrose-soaked cotton ball as a food source. All mosquitoes were given 3 d to mate before blood feeding on a human volunteer twice over 3 d to encourage egg development. Following this, all females were aspirated and transferred to individual 25-mL vials with moist filter paper for 4 d for oviposition. Eggs were counted and allowed to embryonate for 3 d at 28 °C before flooding in aged tap water. Eggs were observed over 2 d for hatching. The hatch rate of eggs was calculated by recording the number of hatched eggs (i.e., egg cap removed) per number of eggs laid by each respective female.

For fecundity estimations, eggs were flooded in trays at a density of 500 larvae/4 L aged tap water and fed daily for 5 d before being sorted into pupation trays. Adults were provided 10% sucrose solution, in the days prior being fed defibrinated sheep’s blood that had been warmed to 37 °C in Petri dishes covered with stretched parafilm that had been rubbed on human skin (Applied Biological Products Management). When adults were 2 to 3 d old, they were offered the bloodmeal on 2 d. Following this, nine random females were allowed to oviposit as described above (see *Assessment of wAlbB2-F4 CI and Fecundity*). Eggs were counted and allowed to embryonate for 3 d before flooding in aged tap water. Eggs were observed over 2 d for hatching. The hatch rate of eggs was calculated by recording the number of hatched eggs (i.e., egg cap removed) per number of eggs laid by each female. All rearing was conducted at 28 °C and 70% humidity with a 6 AM–6 PM light cycle.

### Mass Rearing, Sex Sorting, and Field Delivery.

Supercolonies of ∼12,000 adults with a ∼4:1 (female:male) ratio were housed in BugDorm-4E4545 cages (45 cm^3^ BugDorm) in a sheltered area of a semifield cage at the Tropical Medicine MRF at JCU (62). Colonies were provided access to a 10% sucrose water solution through soaked cellulose sponges refreshed daily. Mass rearing occurred from October 2017 (spring) to May 2018 (autumn), with the temperature within Cairns averaging a daily high of 30 °C and low of 22 °C and an average daily humidity high of 74% and a low of 64%. When daily max temperature failed to reach 26 °C (in winter) a fan heater (Home & Co) was introduced to the colony area (April 2018 until the end of production in May 2018). Eggs weighed into lots of 0.04 g (∼3,000 eggs) and portioned into 50-mL falcon tubes. Each tube was then filled with 40 mL 1-d-old fermented bovine liver solution (CurEase) and allowed to hatch for 3 h before being transferred to rearing trays (26 cm wide, 40 cm long, and 7 cm high; Cambryo), prefilled with 1.7 L tap water. Larvae were fed on a diet of bovine liver powder for 7 d at 28 ± 2° with a 9:15 (day:night) photoperiod. Pupae were subjected to two levels of sex separation on day 8 of hatching using Verily pupal sieves and adult sex separating technology that utilized an industrial vision system and machine learning classifier to compute the probability of the individual being male to achieve very low female contamination (26).

Colonies were run for 16 d from adult eclosion to the removal of the second gonotrophic cycle ovistrip before being destroyed. Females were blood fed defibrinated sheep's blood (Applied Biological Products). Blood was heated in an incubator to 42°C and poured onto the top of the 10-cm–diameter Petri dish lids, and stretched parafilm that had been rubbed on human skin was used to seal in the blood. The blood-filled dishes were then screwed onto a container that had been filled with water heated to 45 °C; the heated water worked to increase the adults feeding time. Blood was introduced to the cage for gonotropic cycle 1 on days 5 and 6 and again for gonotropic cycle two on days 11 and 12. Cages were starved of sucrose solution for 5 h prior to blood feeding to encourage higher feeding rates. Females oviposited on matte waterproof paper lining/ovistrips (Consumable Smart) placed around the inside of a round plastic container (500 mL). Each container was three-fourths filled with tap water and 20 mL rearing water—previously set aside each week when pupating trays were sieved, which encouraged oviposition. Ovistrips were introduced to the cages 24 h after the feeds and were removed after 4 d. The ovistrips were rinsed of dead insects using a pipette filled with tap water. Eggs were allowed to embryonate by laying the ovistrips flat in a sealed plastic container for 48 h. The lid of the container was left off for the first 24 h or until the ovi-strips became dry to the touch, and then they were sealed for storage. Supercolonies were destroyed 16 d after establishment, with cages placed in a freezer to kill adults.

As stated in *Mass Rearing, Sex Sorting, and Field Delivery*, sex separation was a two-step procedure; in the first step, size separating sexually dimorphic pupae using handheld sieves developed by Verily Life Sciences (26). The second step had pupae enclosing into a specialized visual adult sorting technology developed by Verily Life Sciences (26). Adult males were sex-sorted and counted into release tubes containing 10% sucrose. The tubes containing 3-d-old males were then driven 1.5 h into the field early in the morning with most releases occurring between 7 and 11 AM via a Verily customized delivery van that would blow out ∼50 males/site a by following a Google map with predetermined release locations controlling the delivery.

### Molecular Identification of Field-Collected Samples.

All BGS-collected adults were assessed individually for the presence of *w*AlbB2-F4 and *w*Mel *Wolbachia* strains using the DNA extraction and PCR method described in *Molecular Identification of Field-Collected Samples*. Special attention was given to identifying any females carrying the *w*AlbB2-F4 strain, as this could lead to the establishment of *w*AlbB2-F4 population in the landscape and impede the incompatibility of the released males. The BGS-collected adults were separated into males and females and then PCR identified for presence of *Wolbachia* strain using a 96 well nondestructive DNA extraction protocol described in *Molecular Identification of Field-Collected Samples*. Having a nondestructive DNA extraction permitted adult mosquito carcasses to be reassessed for anomalies, as the human error rate in identifying the sex of captured adults is likely much higher than the female contamination rate of the Verily sex sorting pipeline. Additionally, *w*AlbB2-F4 female establishment was monitored by assessing the larvae from the paired ovitraps. Here, five larvae were pooled together to achieve 10% of the total larvae number in the ovitrap (e.g., if 100 larvae are in the ovitrap, then two pools of 5 larva would be assessed). Pooled larvae had DNA extracted before being assessed for the presence of Wolbachia strain.

For the DNA extraction, mosquitoes were placed into 93 wells of a 96-well plate, leaving space for controls (*Wolbachia* strains and DNA extraction controls). DNA was liberated overnight at 55 °C from mosquitoes in sealed 96-well plates in 50 μL lysis buffer (1.0 M NaCl, 0.2 M sucrose, 0.1M Tris ⋅HCl (pH 9.0), 0.05 M ethylenediaminetetraacetic acid, 0.5% sodium dodecyl sulfate containing 1 uL of (20 mg/mL) proteinase K (Sigma-Aldrich AU) per well. Post incubation, 50 μL binding buffer (7.5 M ammonium acetate pH 6) and 50 μL 96% ethanol were added and mixed to make 150 μL. This volume was then aspirated into labeled EPOC filter plates (EPOCH Life Science). The filter plate was then placed in a 96-well 1.1-mL collection plate (Axygen Fisher Scientific), balanced with a second plate of extractions and spun for 1 min at 3,500 rpm. Flow through was discarded, and plates underwent two 50-μL washes (10 mM Tris ⋅ HCl pH 7.5, 80% ethanol) with the first spin for 1 min and the second spin being 5 min to dry the plate—plates were left another 30 min on the bench to dry any excess ethanol on the filter. The plates of mosquito carcases were stored at in a −20-°C freezer and could be used later to confirm morphology, sex, or be re-extracted for genomic DNA.

Since we were releasing wAlbB2 *Ae. aegypti* males into a mosaic of *Aedes* Stegomyia species containing different *Wolbachia* strains, a gel-based PCR was developed to the outer surface protein (wsp) sequence strain variation to distinguish *Ae. aegypti* wild type (no *Wolbachia*) from *Ae. aegypti w*Mel (A lineage *Wolbachia*) and *Ae. aegypti w*AlbB2, while excluding amplification of the local *Ae. notoscriptus* (*w*Noto *Wolbachia*), which shares the same container-inhabiting niche (63). The multiplex PCR utilized the following primers (500 nM): universal reverse primer WSPG514r (5′- CCA TYA AAA YTA GCA CCA TAA GAA CC-3′), with a *w*Mel lineage–specific forward primer WSPM91f (5′-ATA AGA AAG ACA AGA GTG ATT ACA GTC C-3′, band 422 base pairs (bp) and a *w*AlbB2-specific primer WSPB247f55 (5′- CCA ACA ACT GTT GCA AAC AGT GTG G-3′, band 270bp). Additionally, an ribosomal DNA ITS1 primer set was included in the PCR to assess genomic DNA quality. Additionally, the ITS1 products could also be used also distinguish *Ae. aegypti* from *Ae. notoscriptus* and other contaminating mosquito species if required (64). The other PCR components (Bioline MyTaq) were cycled in 20-µL volume containing 1 µL extracted gDNA and incubated for 3 min at 95° then cycled 35 times through 95 °C, 30 s, 55 °C, 30 s, and 72 °C, 30 s before being run in a 1.5% wide minisub agarose gel (BioRad) using four 26-well combes that accommodated the 96 wells along with DNA ladders.

### Suppression Estimates.

Total suppression of *Ae. aegypti* across all three landscapes was measured as an aggregate of relative female trap ratio of the treatments (T1 to T3) to controls (C1 to C3) as counts/trap/day. To assess whether there was a significant downward trend in treated populations relative to control populations, we computed the time series of log relative population as

$$R_{t}=\log\left(\frac{T_{t}}{C_{t}}\right).$$

In constructing this series, *T*_*t*_ is the observed mean trapping rate in the treated landscape for week *t* and *C*_*t*_ is the observed mean trapping rate in the treated landscape for week *t*. The logarithm of the ratio of the time series was used so that *R*_*t*_ exhibited trends that could be considered approximately linear. Linearity under a log transform would be expected if the treated population was undergoing exponential decay (as a result of population suppression) and the control population remained relatively constant. Linear trend in the weekly time series *R*_*t*_ was studied using the nonparametric Theil–Sen estimator (30)—a robust estimate of trend that is insensitive to outliers.

To avoid subjectivity in the comparisons of treatment and control landscapes, we computed Theil–Sen estimates of slope for all possible pairwise comparisons of treatment and control landscapes. *P* values were also calculated, using a one-sided test of significance, since our alternative hypothesis was that there was a significant negative trend in *R*_*t*_ arising from population suppression. Negative trends indicate that population suppression is evident, and statistically significant downward trends can be identified using a 0.05 significance level. As there are simultaneous hypothesis tests, two methods were used to control either the FWER or the FDR. The Bonferroni correction (31) was used to control the probability of making one or more Type-I errors to less than 0.05 and the method of Benjamini and Hochberg (32) for controlling the FDR to be less than 0.05. Controlling the FDR provides a useful alternative to the conservative Bonferroni correction, which has diminished statistical power as the number of hypothesis tests grows in number.

### Monitoring the Suppression Carryover through the Following Season.

A total of 13 adult BGS traps were re-established in each of the three treatment landscapes and one control landscape (C1 Wangan). Trap collections were made weekly for 27 wk from October 23, 2018 to April 30, 2019, and all adult *Ae. aegypti* collected were assessed for the presence of *Wolbachia* strains by PCR (see *Molecular Identification of Field-Collected Samples*).

### Community Engagement.

From the outset of the project, a communication and engagement team strategy was used to assist the initial mosquito survey that involved deployment of 300 GAT traps through the Northern Casssowary Coast urban areas. Initially, CSIRO human ethics approval was obtained for the “Sterile Insect Technology” (026/16), and a stakeholder analysis was undertaken, with a large number of communication and engagement activities used as a result of understanding how best to communicate to these stakeholders and the community. A range of assets and media were utilized to inform the community about the project and a community social license to operate was achieved through a project advisory group (PAG) made up of diverse community leaders including community organizations, local business, local council, and local indigenous representatives. Higher-level monthly meetings were also used to update local and state government health authorities on the trajectory and progress of the project. Final PAG approval for releases to begin were obtained in November 2017. This group met on a monthly basis, and members remained enthusiastic and supportive throughout the life of the project.

## Supplementary Material

Supplementary File

## Data Availability

Field collection data for this IIT experiment has been deposited in a publicly accessible database:Beebe, Nigel (2021): Debug Innisfail data portal version 1. CSIRO. Data collection (https://doi.org/10.25919/3ehh-3q96).
