# Supplementary material for: Releasing incompatible males drives strong suppression across populations of wild and Wolbachia-carrying Aedes aegypti in Australia
Source: Proc Natl Acad Sci U S A. 2021 Oct 4;118(41):e2106828118. doi: 10.1073/pnas.2106828118 (PMC8521666; doi:10.1073/pnas.2106828118)

PNAS
www.pnas.org

Supplementary Information for
Releasing incompatible males drives strong suppression across populations of wild and *Wolbachia* carrying *Aedes aegypti* in Australia

Nigel W. Beebe[a,b]*, Dan Pagendam[b], Brendan J. Trewin[b], Andrew Boomer[b], Matt Bradford[b], Andrew Ford[b], Catherine Liddington[b], Artiom Bondarenco[b] , Paul J. De Barro[b], Joshua Gilchrist[b] Christopher Paton[c], Kyran M. Staunton[c,d], Brian Johnson[e], Andrew J. Maynard[a], Greg J. Devine[e], Leon E. Hugo[e], Gordana Rasic[e], Helen Cook[f], Peter Massaro[g], Nigel Snoad[g], Jacob E. Crawford[g], Bradley J. White[g], Zhiyong Xi[h], and Scott A. Ritchie[c,d]

CORRESPONDING AUTHOR

Nigel Beebe
Email: n.beebe@uq.edu.au

Table S1. Confirmation of bidirectional incompatibility between *w*AlbB2-F4 and *w*Mel *Wolbachia* strains in *Aedes aegypti*.

Table S2. Summary of the *Aedes aegypti* wild type and *w*Mel adult *Ae. aegypti* collections through the following season

Figure S1. Confirmation of *w*AlbB2-F4 *Wolbachia* cytoplasmic incompatibility shown through mating experiments. Bidirectional incompatibility between *w*AlbB2-F4 and *w*Mel is confirmed and only mating between the same *Wolbachia* strains are successful. Cairns wild type represents Queensland wild type *Ae. aegypti*, WB2 is USA-*w*AlbB2-*Ae. aegypti* used for the backcrossing to generate *w*AlbB2-F4 strain. Both wild type and *w*Mel *Ae.* aegypti are currently extant in north Queensland (see Fig. 1).

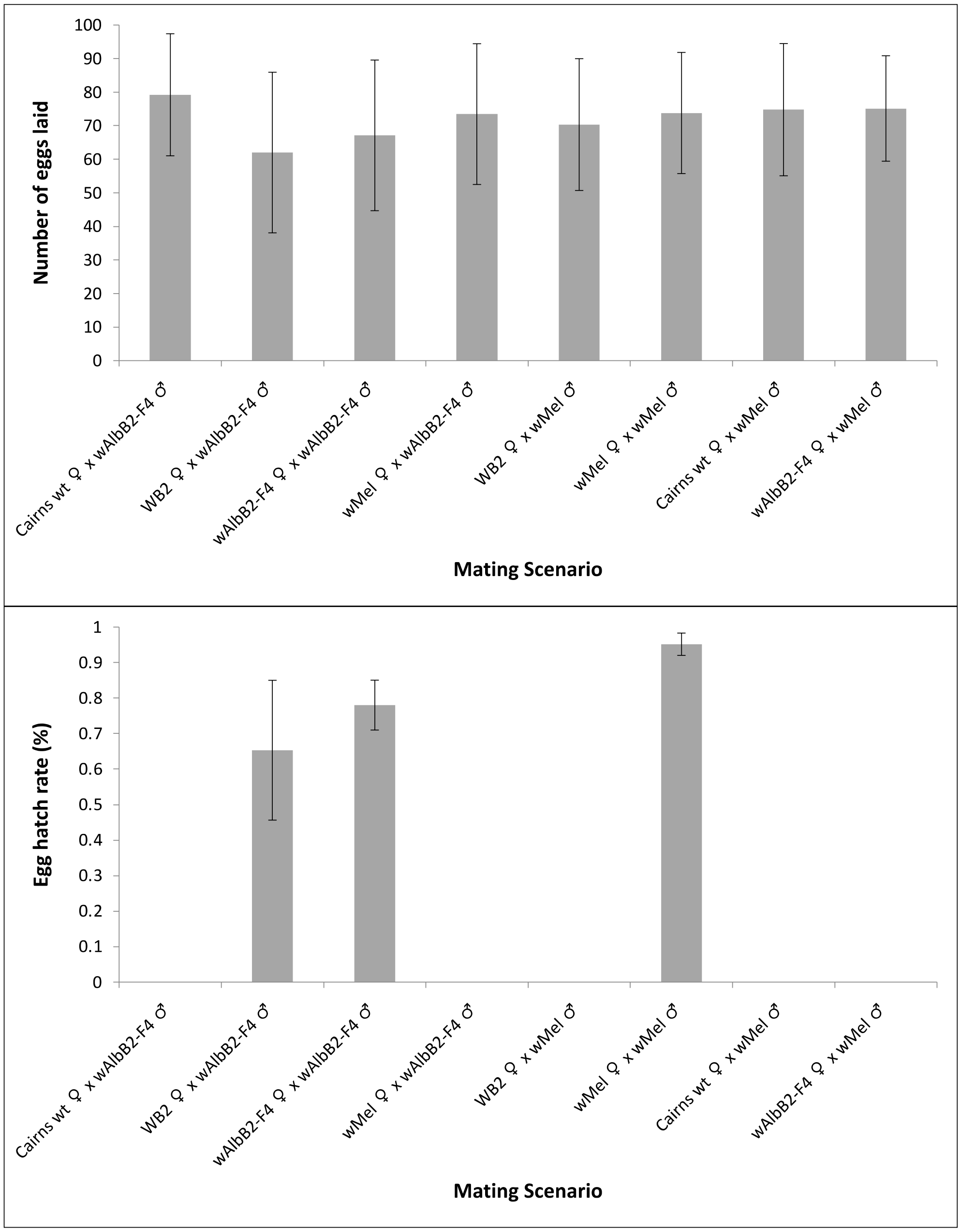

Figure S2. Summary of the wild type and *w*Mel adult *Ae. aegypti* collections from the BGS traps through the following season indicating wild type and *w*Mel-*Ae. aegypti*. The numbers of *Ae. aegypti* collected carrying wMel *Wolbachia* are in blue (*w*Mel+) and the wild type are red (*w*Mel-). The percentage of *w*Mel+ adults collected in the population is represented by the grey line. As very few *Ae. aegypti* were collected in T1-Mourilyan through the following season the data is intermittent. The November 2018 heat wave is indicated by the dashed box.

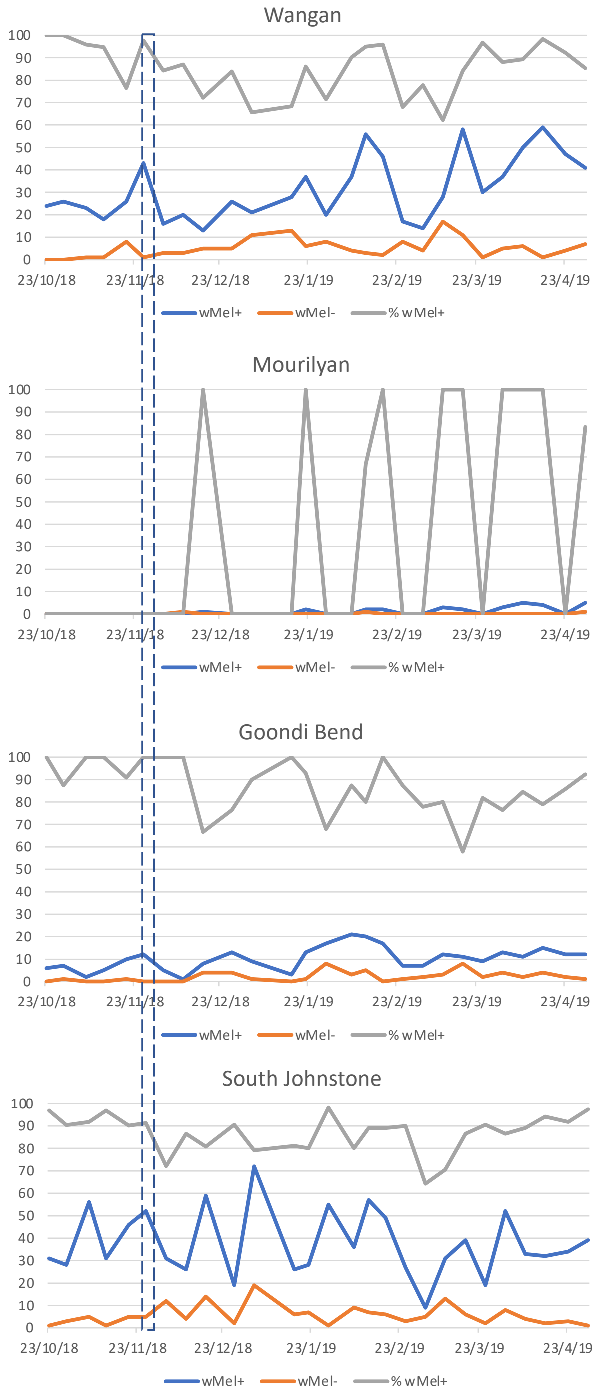

Supplement: Supplementary File [file pnas.2106828118.sapp.pdf]
